# Impact of severe obesity on exercise performance in heart failure with preserved ejection fraction

**DOI:** 10.14814/phy2.14634

**Published:** 2020-11-18

**Authors:** Satyam Sarma, James MacNamara, Sheryl Livingston, Mitchel Samels, Mark J. Haykowsky, Jarett Berry, Benjamin D. Levine

**Affiliations:** ^1^ Institute for Exercise and Environmental Medicine Texas Health Presbyterian Hospital Dallas Dallas TX USA; ^2^ Department of Internal Medicine University of Texas Southwestern Medical Center Dallas Dallas TX USA; ^3^ School of Nursing University of Alberta Edmonton AB USA

**Keywords:** exercise, HFpEF, obesity

## Abstract

**Background:**

Obesity plays an important role in functional impairment in HFpEF. The mechanisms underlying decreased functional capacity in obese HFpEF are not clear. We assessed the cardiac and peripheral determinants of exercise performance in HFpEF patients with class 2 obesity in the upright position, representative of posture when performing functional activities.

**Methods and Results:**

Thirty‐two HFpEF patients were divided into two groups by presence of class 2 obesity (C2, BMI ≥ 35 kg/m^2^, *n* = 14) and non‐C2 (BMI < 35 kg/m^2^, *n* = 18). Participants performed a bout of submaximal exercise followed by incremental stages of treadmill exercise to determine peak aerobic power (peak VO_2_). Peak VO_2_ and Ve/VCO_2_ were measured using Douglas bags while cardiac output (Qc) and stroke volume (SV) were measured by acetylene rebreathing. The C2 group were younger than the non‐C2 group (67 ± 6 versus 73 ± 6 years; *p* = .009). Comorbid condition burden was similar between groups. Peak VO_2_ indexed to body mass was not significantly different between groups. Absolute peak VO_2_ was higher in the C2 group secondary to a larger peak Qc (14.3 versus 11.0 L/min; *p* = .012). SV reserve was also higher in the C2 group (72 versus 49%; *p* = .038).

**Conclusion:**

HFpEF patients with severe obesity had similar cardiorespiratory fitness compared to patients with lower BMI with similar comorbidity burden. Absolute VO_2_ was actually higher in the severely obese driven by larger Qc and SV reserve arguing against significant effects from obesity per se on aerobic performance. The presence of a larger “cardiac engine” may offer potential for fat‐loss strategies to improve impairments in functional capacity in obese patients with HFpEF.

## Graphical abstract

Obesity is linked to decreased functional capacity in in patients with heart failure with preserved ejection fraction (HFpEF).The mechanisms responsible for decreased functional capacity are unclear. Our study demonstrated HFpEF patients with severe obesity have higher absolute VO_2_, a result of larger cardiac output and stroke volume reserve, highlighting a potential role for fat loss strategies to improve impairments in functional capacity.

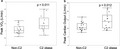

## 1 INTRODUCTION

Obesity is a common comorbid condition in heart failure with preserved ejection fraction (HFpEF) and is present in approximately 40%–50% of patients (Haass et al., (2011); Shah et al., 2013). Particularly in patients with class 2 obesity or higher (35 kg/m^2^), obesity is an important risk factor for all‐cause mortality, HF hospitalization, and impaired functional capacity (Dalos et al., 2016; Haass et al., 2011). Mechanistic studies suggest alterations in right ventricular function and pericardial constraint with increasing body mass index (BMI) lead to larger rises in pulmonary capillary wedge pressures with exertion (Obokata et al., 2017). These pathologic changes may represent adaptations uniquely attributable to obesity, suggesting the presence of a distinct obesity HFpEF phenotype (Kitzman & Shah, 2016).

Assessment of exercise performance in obesity can be influenced by several factors. Obesity is commonly associated with comorbid conditions (e.g., diabetes, sleep apnea) that are known to impart unfavorable changes in cardiac structure and function in HF. These common conditions present challenges in isolating the independent impact of obesity on cardiac performance during exercise (Beitler et al., 2014; O'Connor et al., 2015). From an anthropomorphic standpoint, indexing exercise performance parameters to very large body weights and BMI can overemphasize the effects of scaling (Nevill et al., 1992; Vanderburgh & Katch, 1996). Finally, exercising in the supine position exaggerates differences in exercise capacity among the obese through increased loss of mechanical advantage due to larger leg mass and exacerbation of ventilatory constraints common in obesity (Babb et al., 2002; Too, 1990).

The aim of our study was to characterize the impact of obesity on cardiac and peripheral determinants of exercise performance and cardiorespiratory fitness in HFpEF patients measured in the upright position, a more representative postural state for patients performing activities of daily living. We divided patients into two groups by the presence of class 2 obesity (C2, BMI > 35 kg/m^2^ versus non‐C2, BMI < 35 kg/m^2^) and hypothesized there would be no group differences in peak exercise aerobic power (peak VO_2_), cardiac output (Qc), or stroke volume reserve.

## 2 METHODS

HFpEF patients were recruited from a university cardiology clinic. In addition, exercise performance data in 11 subjects previously reported was used to supplement the present analysis to increase power for group comparisons (Bhella et al., 2011). There was an even distribution of the 11 into both groups: six had BMI < 35 kg/m^2^ and five were >35 kg/m^2^. The Institutional Review Boards (IRB) of the University of Texas Southwestern Medical Center and Texas Health Resources approved all study procedures. Subjects were invited to participate if they: (1) were older than 60 years of age; (2) had been hospitalized previously for HF; (3) had evidence of pulmonary congestion by chest x‐ray or elevated cardiac filling pressures (pulmonary capillary wedge or left ventricular end‐diastolic pressures >16 mmHg by heart catheterization); and (4) LV ejection fraction >50%. HFpEF subjects were excluded for: (1) body mass index > 45 kg/m^2^, (2) eGFR < 30 ml/min/m^2^, (3) severe chronic obstructive pulmonary disease (COPD), (4) chronic atrial fibrillation, (5) constrictive or restrictive cardiomyopathy, (6) severe valvular disease or history of valvular surgery, or (7) if they were unable to perform exercise testing. Diuretic and blood pressure regimens needed to be stable for at least 3 months prior to enrollment.

### 2.1 Exercise testing

Subjects performed upright exercise at a submaximal intensity (≈50% peak VO_2_ determined from a previous maximal exercise test) for 5 min followed by a modified Astrand–Saltin incremental treadmill protocol to exhaustion. Measures of ventilatory gas exchange were made by use of the Douglas bag technique both at rest and during exercise (Arbab‐Zadeh et al., 2004). Gas fractions were analyzed by mass spectrometry (Marquette MGA 1100), and ventilatory volume was measured by a Tissot spirometer. Peak VO_2_ was defined as the highest oxygen uptake measured over a 30‐s period. Cardiac output and SV were measured using a modified acetylene gas rebreathing technique (Hardin et al., 2020) and AVO_2_ difference was calculated from cardiac output and peak VO_2_. Stroke volume reserve was defined as the percentage change in SV from rest to submaximal exercise. Blood pressure was measured during exercise using an ECG gated sphygmomanometer (Tango; SunTech Medical, NC, USA).

### 2.2 Statistical analysis

Statistical analysis was performed using commercially available software (Prism, GraphPad San Diego, CA). All reported variables are presented as means with standard deviations. Student's *t* test were used to test differences between groups. A *p* < .05 was considered statistically significant.

## 3 RESULTS

Fourteen subjects (41%) were in the C2 group (BMI: 39.3 ± 2.4 kg/m^2^) while 18 subjects were in the non‐C2 group (30.8 ± 3.3 kg/m^2^). The C2 group were younger than the non‐C2 group (67 ± 6 versus 73 ± 6 years; *p* = .009). No significant difference was found between groups for hypertension, diabetes, sleep apnea, or medication usage (Table 1). In general, there were no differences in NYHA heart failure class between groups but there tended to be more NYHA class I functional capacity in the non‐C2 group. There were no differences in echocardiographic markers of diastolic relaxation (Table 2).

**Table 1:** Demographic and exercise performance variables

	Non‐class 2 obesity (*n* = 18)	Class 2 obesity (*n* = 14)	*p*
Group characteristics			
Age (years)	73 ± 6	67 ± 6	0.009
Men, *N* (%)	8 (44%)	5 (36%)	NS
BMI (kg/m^2^)	30.8 ± 3.3	39.3 ± 2.4	<0.001
Weight (kg)	85.6 ± 15.8	108.4 ± 12.3	<0.001
Hypertension, *N* (%)	18 (100)	14 (100)	NS
Diabetes, *N* (%)	8 (44)	8 (57)	0.49
Sleep apnea, *N* (%)	10 (56)	9 (64)	0.81
NYHA Class			
I, *N* (%)	4 (22)	1 (7)	0.45
II, *N* (%)	5 (28)	5 (36)	
III, *N* (%)	9 (50)	8 (57)	
Medications			
Beta‐blocker (%)	11 (61)	12 (86)	0.13
Loop diuretic (%)	15 (83)	13 (93)	0.44
ACE inhibitor (%)	14 (78)	10 (71)	0.69
Exercise parameters			
Peak VO_2_ (L/min)	1.10 ± 0.31	1.51 ± 0.54	0.011
Peak VO_2_ (mL/kg/min)	12.9 ± 2.8	13.8 ± 3.9	0.45
Peak HR (bpm)	122 ± 21	135 ± 23	0.12
Peak mean arterial pressure (mmHg)	114 ± 12	115 ± 16	NS
Peak RER	1.00 ± 0.10	1.01 ± 0.07	NS
Resting cardiac output (L/min)	4.2 ± 0.9	4.9 ± 1.8	0.13
Peak cardiac output (L/min)	11.0 ± 2.4	14.3 ± 4.5	0.012
Peak cardiac index (L/min/m^2^)	5.6 ± 1.4	6.4 ± 2.1	0.20
Rest stroke volume (ml)	59 ± 15	60 ± 19	NS
Peak stroke volume (ml)	91 ± 18	105 ± 21	0.059
Stroke volume reserve (%)	49 ± 21	72 ± 39	0.038
Peak AVO_2_ difference (%)	10.1 ± 2.4	10.7 ± 3.1	NS
Peak systemic vascular resistance (dynes/cm^5^)	852 ± 192	691 ± 221	0.042
Peak minute ventilation (L/min)	46.3 ± 13.1	54.3 ± 13.6	0.11
Ve/VCO_2_	42.9 ± 6.2	38.7 ± 7.6	0.07

**Table 2:** Echocardiography diastolic parameters

	Non‐class 2 obesity	Class 2 obesity	*p*
Mean e’ TDI (cm/s)	7.1 ± 1.4	7.2 ± 1.9	0.94
E wave (cm/s)	87 ± 38	80 ± 21	0.59
A wave (cm/s)	86 ± 34	103 ± 30	0.24
E/A ratio	1.07 ± 0.51	0.81 ± 0.20	0.12
E/e’ ratio	12.7 ± 6.6	11.6 ± 3.3	0.62

**Figure 1:**
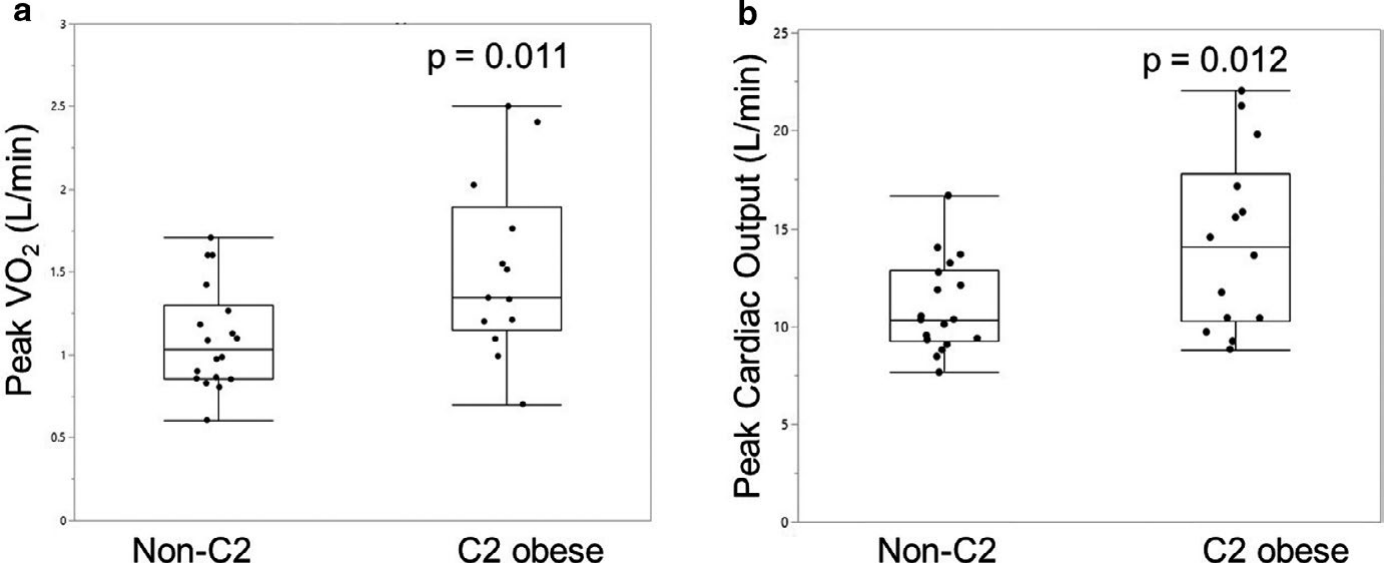
Distribution of peak VO_2_(a) and peak cardiac output (b) in HFpEF patients without class 2 obesity (non‐C2) and with class 2 obesity (C2). Box plot shows median and interquartile range. Both peak VO_2_and cardiac output were significantly higher in HFpEF patients with class 2 obesity or greater

Peak VO_2_ (L/min) was higher in the C2 group compared to the non‐C2 group (1.51 ± 0.54 versus 1.10 ± 0.31, *p* = .011) with no significant difference between groups for VO_2_ indexed to body mass (13.8 ± 3.9 versus 12.9 ± 2.8 ml kg^‐1^ min^‐1^; *p* = .45) (Table 1). There were marked differences in peak cardiac output and cardiac output reserve between groups (11.0 ± 2.4 versus 14.3 ± 4.5 L/min; *p* = .012 C2 versus NC2, respectively). Figure 1 shows the distribution of individual peak VO_2_ and cardiac output from both groups. Cardiac output reserve from rest to peak exercise was 30% higher in the C2 group (6.8 ± 2.1 versus 9.4 ± 3.2 L/min; *p* = .011). Both peak heart rate and stroke volume were numerically higher in the C2 group while SV reserve was significantly higher in the C2 group. No significant difference was found between groups for peak AVO_2_ difference or ventilation, though there was a trend toward improved ventilatory efficiency, measured by Ve/VCO_2_ ratio, in the C2 group (42.9 ± 6.2 versus 38.7 ± 7.6; *p* = .07).

## 4 DISCUSSION

The major new finding of this study is that severe obesity has no significant negative impact on cardiorespiratory parameters of exercise performance in HFpEF patients with relatively balanced burdens of comorbid conditions. Absolute peak VO_2_ and cardiac output, both non‐weight‐based parameters, were significantly higher in patients with class 2 obesity compared to non‐C2 HFpEF patients consistent with their larger body size. Peak heart rates and stroke volumes were similar while stroke volume reserve was significantly higher arguing against significant exercise limiting chronotropic, inotropic, and lusitropic impairments attributable to severe obesity alone.

Given the high prevalence of obesity among HFpEF patients, there is a paucity of studies addressing the effect of obesity on disease severity and progression. The analysis of the I‐PRESERVE study demonstrated a U‐shaped mortality curve by BMI strata with both BMI < 23.5 kg/m^2^ and >35.0 kg/m^2^ associating with higher mortality (Haass et al., 2011). These findings were in contrast to a smaller, community‐based cohort study which found obese HFpEF patients (average BMI of 37.7 kg/m^2^) actually had improved survival after adjustment for concomitant comorbid conditions, likely a result of better ventricular and vascular function compared to HFpEF patients with renal dysfunction and diabetes (Mohammed et al., 2012). Finally, the largest study to date characterizing the hemodynamic effects of obesity during exercise described a distinct profile of increased pulmonary vascular resistance, higher mean pulmonary arterial pressure, and evidence for right ventricular dysfunction with elevated right atrial to pulmonary capillary wedge pressure ratio as well as left ventricular septal flattening in obese patients with HFpEF (Obokata et al., 2017). The average BMI reported for the obese group in this study was 40.8 kg/m^2^, similar to our cohort of class 2 obesity HFpEF patients, but the prevalence of baseline comorbid conditions was skewed compared to our present study. For example, rates of sleep apnea and diabetes were significantly higher in their obese HFpEF subjects. In addition, exercise testing in the study was performed in the supine position which may have exaggerated exercise limitations in the obese group and may explain the lack of an obesity effect in our study in which all exercise testing was done upright.

Exercising in the supine position can confer several disadvantages to exercise performance compared to upright posture. This difference is primarily the result of two factors––changes in respiratory mechanics and cycling economy. There is a distinct lower limb mechanical disadvantage with cycling in the supine position resulting in earlier time to fatigue compared to semi‐recumbent and upright exercise (Egana et al., 2010), limitations that may be particularly exacerbated in obese individuals due to larger leg mass. The supine position can also reduce lung volumes and worsen restrictive pulmonary physiology that is common in obese individuals. The larger chest wall mass reduces functional vital capacity and significantly increases end‐expiratory gastric and esophageal pressures resulting in increased work of breathing (Steier et al., 2014). Obesity is also associated with lower arterial oxygen saturations, particularly in the supine position that can be improved after weight loss (Hakala et al., 2000). Although our cross‐sectional study was limited in not assessing these mechanistic factors, these aforementioned pathophysiologic pathways highlight the potential impact of body position on assessing exercise performance in obese individuals.

In addition to exercise body position, scaling strategies to “index” variables can also exaggerate exercise limitations in obesity. Peak VO_2_, for example, is often indexed to body mass, typically reported as ml/kg/min. While this provides important information on a patient's functional capacity in terms of aerobic power to body size mismatch, many obese patients have higher absolute VO_2_ compared to nonobese patients. In this respect, we observed a 0.4 L/min higher VO_2_ in obese HFpEF patients, a result of a nearly 3 L/min higher cardiac output at peak exercise. The higher absolute peak cardiac output and VO_2_ is likely an effect of obesity‐related increases in fat‐free body mass and perhaps increases in plasma volume (Simone et al., 1997). With exercise, mobilization of blood and plasma volume as venous return contributes to increases in stroke volume while higher amounts of fat‐free body mass, particularly in the lower body as an adaptation to chronic carrying of large weight loads, leads to higher oxygen requirements. In addition, the younger age (by 6 years) of the HFpEF patients with severe obesity may have also contributed slightly to higher peak VO_2_.

Our findings are in line with the RELAX trial ancillary study of exercise in HFpEF and obesity (Reddy et al., 2019). While relative scaled VO_2_ was lower in obese HFpEF patients, the absolute unscaled VO_2_ was higher (1.32 versus 1.00 L/min; *p* < .001). Cardiac output, stroke volume reserve, or AVO_2_ difference were not reported in the ancillary study. Our findings that HFpEF patients with obesity have higher stroke volume and cardiac output reserve (i.e., a larger cardiac “engine”) and absolute VO_2_ may explain why weight loss has been particularly successful in improving functional capacity (relative peak VO_2_) and exercise tolerance (Kitzman et al., 2016). Although our results are based on a relatively small sample size, the distribution of comorbid conditions is similar to those reported from larger studies with the exception of renal dysfunction.

In conclusion, severe obesity (BMI > 35 kg/m^2^) in HFpEF patients had no negative effect on absolute peak VO_2_ and in general was associated with improved cardiovascular response to upright exercise with higher peak heart rate, stroke volume, and stroke volume reserve than HFpEF subjects with less severe obesity. The presence of a larger “cardiac engine” may offer a basis for pursuing fat‐loss strategies to improve impairments in functional capacity in obese patients with HFpEF.

## CONFLICTS OF INTEREST

None.

## AUTHOR CONTRIBUTION

S.S. designed, acquired, and analyzed the primary data and wrote the manuscript. J.P.M assisted with data analysis and manuscript editing. S.L. and M.S. were involved in data acquisition. M.J.H, J.B., and B.D.L provided critical feedback on the manuscript. B.D.L. acquired funding for the study and was involved in the conceptual design of the study.

## ETHICAL STATEMENT

This study conformed to the Declaration of Helsinki and was approved by the Institutional Review Boards (IRB) of the University of Texas Southwestern Medical Center and Texas Health Resources

## Data Availability

The data that support these findings are available upon reasonable request from the corresponding author.
